# Computational insights into the role of α-strand/sheet in aggregation of α-synuclein

**DOI:** 10.1038/s41598-018-37276-1

**Published:** 2019-01-11

**Authors:** Anand Balupuri, Kwang-Eun Choi, Nam Sook Kang

**Affiliations:** Graduate School of New Drug Discovery and Development, Chungnam National University, Daejeon, 305-764 Republic of Korea

## Abstract

The α-synuclein is a major component of amyloid fibrils found in Lewy bodies, the characteristic intracellular proteinaceous deposits which are pathological hallmarks of neurodegenerative diseases such as Parkinson’s disease (PD) and dementia. It is an intrinsically disordered protein that may undergo dramatic structural changes to form amyloid fibrils. Aggregation process from α-synuclein monomers to amyloid fibrils through oligomeric intermediates is considered as the disease-causative toxic mechanism. However, mechanism underlying aggregation is not well-known despite several attempts. To characterize the mechanism, we have explored the effects of pH and temperature on the structural properties of wild-type and mutant α-synuclein using molecular dynamics (MD) simulation technique. MD studies suggested that amyloid fibrils can grow by monomer. Conformational transformation of the natively unfolded protein into partially folded intermediate could be accountable for aggregation and fibrillation. An intermediate α-strand was observed in the hydrophobic non-amyloid-β component (NAC) region of α-synuclein that could proceed to α-sheet and initiate early assembly events. Water network around the intermediate was analyzed to determine its influence on the α-strand structure. Findings of this study provide novel insights into possible mechanism of α-synuclein aggregation and promising neuroprotective strategy that could aid alleviate PD and its symptoms.

## Introduction

The α-sheet is an atypical secondary structure in proteins which is also recognized as pleated sheet or polar pleated sheet. This structure is formed by regular hydrogen bonding between adjacent α-strands and was firstly proposed by Pauling and Corey in 1951^1–3^. In contrast with α-helix and β-sheet structures, the α-sheet residues are not constrained to a specific region of dihedral angles. The α-strand consists of alternating dihedrals in the right-handed (α_R_) and left-handed (α_L_) helical regions of Ramachandran plot. Even though α-sheet is not commonly witnessed in natural proteins, it is hypothesized to play a central role in various amyloid diseases^4^. The α-sheet is proposed as a probable intermediate state in the formation of amyloid fibrils on the basis of molecular dynamics (MD) simulations studies. This structure is considered as toxic conformer in amyloid diseases. The α-sheet structure has been observed for lysozyme, prion proteins, β2-microglobulin and transthyretin. These peptides and proteins are involved in many protein misfolding and amyloid diseases^4,5^. They undergo a conformational change from predominantly random coil or α-helix structures to the β-sheet structures which are largely present in amyloid fibrils. Peptide plane flipping mechanism has been reported for direct α-sheet and β-sheet interconversion^6,7^. Apart from MD simulations, X-ray crystallography structures of designed peptides have confirmed α-sheets. Furthermore, several experimentally determined crystal and NMR structures in Protein Data Bank (PDB) have been found to adopt α-strand conformation^4^. Recent experimental studies have reported that designed anti-α-sheet peptides preferentially bind to toxic amyloid precursor (intermediate α-sheet conformer) as compared to non-aggregated, nontoxic species or mature fibrils and inhibit aggregation of amyloid proteins^8–11^. These peptides provide evidence that α-strands and α-sheet are not just computational or theoretical artifacts and they do indeed exist.

The α-synuclein is principal component of numerous protein aggregates known as Lewy bodies and Lewy neurites. Although Lewy bodies are major pathological hallmarks of Parkinson’s disease (PD), α-synuclein is associated with a number of neurodegenerative diseases known as synucleinopathies^12^. This includes Lewy body variant of Alzheimer’s disease (LBVAD), PD dementia (PDD), neurodegeneration with brain iron accumulation type 1 (NBIA1), pure autonomic failure, Down’s syndrome, complex of Guam and diffuse Lewy body disease (DLBD)^13–21^. The α-synuclein is a protein of 140 residues devoid of cysteine and tryptophan. Primary structure of α-synuclein is usually divided into three distinct regions: N-terminal (residues 1–60), central region (residues 61–95) and C-terminal (residues 96–140). N-terminal is amphipathic region while C-terminal mainly consists of acidic residues^22^. Most hydrophobic central region which is also termed as non-amyloid-β component (NAC), folds into a β-sheet secondary structure and plays critical role in both the aggregation and cytotoxicity^23^. Prior studies suggested that α-synuclein fibril core comprise of residues 30–110^24,25^. However, it was found that core domain of α-synuclein could be even shorter and comprise of residues 71–82^26–30^.

α-synuclein is an intrinsically disordered protein that may undergo dramatic structural changes to form amyloid fibrils. Several conformational states for this protein have been observed. Aggregation process from monomers to amyloid fibrils through oligomeric intermediates is considered as the disease-causative toxic mechanism^24^. Various intrinsic and extrinsic factors which could either increase or decrease the rate of α-synuclein aggregation *in vitro* are known. These factors include temperature, pH, point mutations (A30P, E46K and A53T), NAC region mutations, S-129 phosphorylation, N-terminal acetylation, organic solvents, metal ions and salts^12,31–41^. However, mechanism underlying aggregation is not well-known despite several attempts. Previous studies have suggested the role of a partially folded intermediate in aggregation and fibrillation of α-synuclein^31,42^. Recently, β-hairpin formation in the region 38–53 (part of N-terminal) was proposed to be responsible for α-synuclein aggregation^22^. However, the role of α-strand/sheet in α-synuclein aggregation has not been studied yet.

As α-sheet structure was found to be intermediate state in the formation of fibrils for various amyloid proteins^4,5^. In the present article, we investigated the role of α-strand/sheet in the aggregation of α-synuclein. To characterize the mechanism, we have explored the effects of pH and temperature on the structural properties of wild-type and mutant α-synuclein using molecular dynamics (MD) simulation technique. Furthermore, we studied the effect Ser-129 phosphorylation and N-terminal acetylation. Here, we described the structural and dynamic changes in α-synuclein and proposed a model for aggregation and fibrillation of this protein. MD studies suggested that amyloid fibrils could grow by monomer. Conformational transformation of the natively unfolded protein into α-strand/sheet intermediate could be accountable for fibrillation. Our results provide structural insights which could be utilized for the development of anti-PD drugs targeting the NAC region of α-synuclein.

## Materials and Methods

### Structures of human α-synuclein and mutants

Several structures of α-synuclein have been determined using X-ray, NMR and electron microscopy techniques, yet most of the structures are available for small fragments of α-synuclein (http://www.rcsb.org/). Merely, three NMR structures exist for full-length α-synuclein. Out of these, Protein Data Bank (PDB) codes 1XQ8^43^ and 2KKW^44^ are micelle-bound α-synuclein structures. Recently, third NMR structure (PDB 2N0A)^45^ was reported for a pathogenic fibril of full-length human α-synuclein. We have used chain A of PDBs 2N0A and 2KKW for our study. Structures for the mutants were generated by computationally mutating the corresponding amino acid residue in the wild type NMR structure (PDB 2N0A)^45^. Mutagenesis Wizard of PyMOL (The PyMOL Molecular Graphics System, Schrödinger, LLC) was used for creating the point mutation. The resulting mutant structure was energy-minimized prior to MD simulation. Residue Ser-129 was phosphorylated using “Build and Edit Protein” module of the Discovery Studio 2017 R2 (BIOVIA, San Diego: Dassault Systèmes).

### Molecular dynamics simulations

All the MD simulations were performed using GROMACS 5.1.3 package^46^ with CHARMM27 force field^47^. Chain A of PDBs 2N0A^45^ and 2KKW^44^ were employed as the starting point for the simulations. MD simulation were performed at neutral and low pH (protonated His, Asp and Glu)^5,48,49^. High temperature is reported to accelerate unfolding and folding process without affecting the overall pathway^5,50^. Accordingly, simulations were carried out at high temperature (498 K) besides 300 K. Simulation were performed with periodic boundary conditions at constant temperature, pressure and fixed protonation states. A cubic box with TIP3P water molecules^51^ was used to solvate the protein. Depending upon the protonation state of protein, appropriate number of counter-ions (Na^+^ and Cl^−^) were added to the system to maintain neutrality. System was then minimized using 50,000 steps of steepest descent algorithm to ensure that the system has no steric clashes, inappropriate geometry or and structure distortions. This was followed by 100 ps NVT (constant number of particles, volume, and temperature) and 1000 ps NPT (constant number of particles, pressure and temperature) equilibrations. Temperature was maintained using the v-rescale (modified Berendsen thermostat)^52^ whereas pressure was maintained at 1 atm using the Parrinello-Rahman barostat^53^. The LINCS algorithm^54^ was employed to constrain bond lengths. Long-range electrostatic interactions were treated with particle mesh Ewald summation method^55^ and a cutoff of 12 Å was used for short-range interactions. Production runs were performed for 20 ns. The integration time step was set to 2 fs and the trajectory coordinates and energies were saved at 10 ps intervals. Built-in programs of GROMACS software package^46^ were employed for the analysis.

### Computation of dihedral angles

The dihedral angles were obtained using gmx rama module of GROMACS software package^46^. This module selects the φ/ψ dihedral combinations from topology file and computes these as a function of time. Afterwards, in house *R* scripts were used to identify residues which satisfies α_R_ and α_L_ conformations criteria as well as number of such conformations. A residue was classified in α_R_ conformation if its dihedral angles were within −180 < φ < 0, −180 < ψ < 0 i.e. (−,−) while, if the dihedral angles were within 0 < φ < 180, 0 < ψ < 180 i.e. (+, +), it was categorized in α_L_ conformation. This is in accordance with a previous report on α-strand^4^.

### Topological water network analysis

Water molecules linked by hydrogen bonds form different types of hydrogen-bonded cyclic water-ring networks. Potential functions considered here involve a rigid TIP3P water model. The interactions between water molecules are commonly modeled with Lennard-Jones and Coulomb potentials^51^. The interaction potential energy between water molecules *a* and *b* is expressed by the following equation:

$${\rm{v}}(a,b)=\sum _{i}^{on\,a}\sum _{j}^{on\,b}\frac{{q}_{i}{q}_{j}{e}^{2}}{{r}_{ij}}+\frac{A}{{{r}_{oo}}^{12}}-\frac{C}{{{r}_{oo}}^{6}}$$

Where *r*_*oo*_ denotes the distance between oxygen atoms. The electrostatic attraction is expressed as a coulombic force between two charges *q*_*i*_ and *q*_*j*_ with a distance *r*_*ij*_ between them. The van der Waals interaction is expressed as a function that simultaneously takes attraction and repulsion. Parameter *A* is the repulsive force of *i* and *j* while parameter *C* represents attraction and is an inherent value according to the type of the atom. The parameters *A* and *C* were selected to produce reasonable structural and energetic results for liquid water. The value of parameters are as follows:

$$\begin{array}{c}A=582,000\,{\rm{kcal}}\,{{\rm{\AA }}}^{12}{{\rm{mol}}}^{-1}\\ C=595{\rm{kcal}}\,{{\rm{\AA }}}^{6}{{\rm{mol}}}^{-1}\\ {q}_{i}=-\,0.834e,{q}_{j}=0.417e\end{array}$$

We selected the energy criterion of −2.25 kcal mol^−1^ to determine the hydrogen bond between water molecules as this value correlates with the minimum value of the pair-energy distribution of potential^51^.

## Results and Discussion

α-synuclein is intrinsically disordered (natively unfolded) protein but how it is transformed into the highly organized fibrils is not well understood. Experimental studies demonstrated that α-synuclein fibrillogenesis occurs in the similar way as β-amyloid (Aβ) fibrillogenesis by a multistep, nucleation-dependent polymerization process^56^. It requires seeding by an ordered nucleus, followed by the growth of oligomers through incorporation of further monomers. The first step involves primary nucleation that is the formation of a fibril nucleus from monomers. The formed nucleus grows during the fibril elongation step to produce mature fibrils, which subsequently catalyze the generation of new aggregates by secondary pathways. Generation of new aggregates by secondary pathways (fibril fragmentation or surface-catalyzed nucleation) are dependent on the concentration of existing fibrils. Secondary pathways lead to the formation of a new fibril nucleus which dissociates mature fibril in the final step and participate in polymerization process. Primary nucleation is a slow process while nucleation through secondary pathways is much more rapid^57–59^.

**Figure 1:**
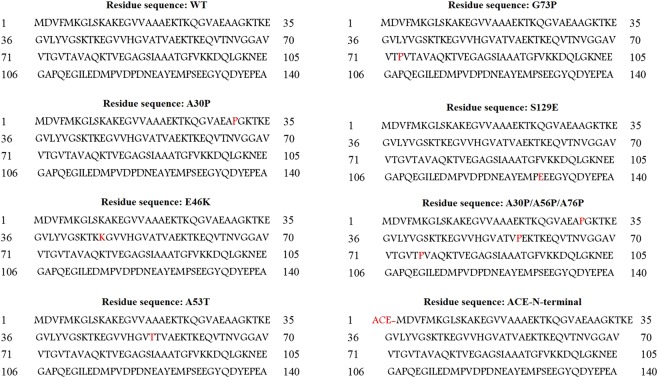
Residue sequences for the simulated systems.

Here, we have explored the possibility of α-strand formation in α-synuclein and the apparent role of α-strand/sheet in the aggregation and fibrillation of this protein. PDBs 2N0A (fibril state) and 2KKW (micelle-bound state) which represent two different conformations of α-synuclein were selected for this study. To examine the α-strand formation, we performed MD simulations of wild type (WT) α-synuclein monomer at neutral and low pH at physiological (300 K) and high temperature (498 K). Furthermore, simulations of A30P, E46K, A53T, A30P/A56P/A76P and G73P variants were carried out to investigate the effect of these mutations on the α-strand formation. Besides, phosphorylation effect was explored by phosphorylation-mimicking mutation S129E as well as by phosphorylating Ser-129^39^. Table 1 summarize the MD simulations performed in the present study. Residue sequences for the simulated systems are displayed in the Fig. 1. We also characterized the probable reasons for the α-strand formation. At the end, aggregation mechanism of α-synuclein on the basis of α-strand/sheet structure is proposed.

**Table 1:** MD simulations carried out in the current study. The aggregated simulation time is 280 ns.

Simulated system	Type	Counter ions	Temperature	pH	MD run
**1**	2N0A, WT	9 Na^+^	300 K	Neutral	20 ns
**2**	2N0A, WT	9 Na^+^	498 K	Neutral	20 ns
**3**	2N0A, WT	16 Cl^−^	300 K	Low	20 ns
**4**	2N0A, WT	16 Cl^−^	498 K	Low	20 ns
**5**	2N0A, Mutant (A30P)	9 Na^+^	300 K	Neutral	20 ns
**6**	2N0A, Mutant (A53T)	9 Na^+^	300 K	Neutral	20 ns
**7**	2N0A, Mutant (E46K)	7 Na^+^	300 K	Neutral	20 ns
**8**	2N0A, Mutant (A30P/A56P/A76P)	9 Na^+^	300 K	Neutral	20 ns
**9**	2N0A, Mutant (G73P)	9 Na^+^	300 K	Neutral	20 ns
**10**	2N0A, Mutant S129E	10 Na^+^	300 K	Neutral	20 ns
**11**	2N0A, Ser(P)-129	10 Na^+^	300 K	Neutral	20 ns
**12**	2N0A, ACE-N-terminal	10 Na^+^	498 K	Neutral	20 ns
**13**	2KKW, WT	9 Na^+^	498 K	Neutral	20 ns
**14**	2KKW, WT	16 Cl^−^	498 K	Low	20 ns

### Definition of the α-strand structure

**Figure 2:**
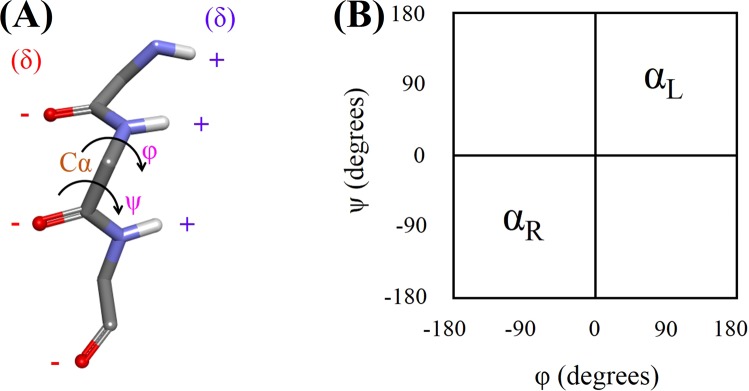
(**A**) The α-strand structure displaying the backbone dihedral angles and arrangement of the partial charges. Side chain and non-polar hydrogen atoms of the residues are not displayed for clarity. Phi (φ) is C-N-Cα-C dihedral angle while Psi (ψ) is N-Cα-C-N dihedral angle. (**B**) Ramachandran or (φ,ψ) plot showing the right-handed (α_R_) and left-handed (α_L_) helical regions.

The α-strand is defined by an alternation of amino acid residues in helical, α_R_ and α_L_ conformations. Bifurcated hydrogen bonding between two nearby α-strands forms an α-sheet. Previous studies suggested various average (φ, ψ) for the α-sheet structures^4,6^. In the present study, we analyzed the occurrence of α-strand structure in α-synuclein. Herein, successive residues were considered to adopt α-strand conformation if they exist in α_R_α_L_α_R_ or α_L_α_R_α_L_ conformations. In an α-strand, all the backbone carbonyl groups are oriented in the same direction on one side of the strand whereas all the backbone amino groups are oriented in the same direction on the reverse side of the strand. As a result, one edge of the α-strand is negatively charged while the opposite edge is positively charged. Figure 2 shows the α-strand structure, backbone dihedral angles, arrangement of the partial charges and Ramachandran or (φ,ψ) plot for right-handed (α_R_) and left-handed (α_L_) helical regions.

### Occurrence of α-strand in the region 72–74

Amyloidogenic NAC region is crucial for aggregation and cytotoxicity of α-synuclein. This region is known to fold into a β-sheet secondary structure^60^. A hydrophobic stretch of 12 amino acids (residues 71–82) in the middle of NAC region is vital for filament assembly. Aggregation and neurotoxicity of α-synuclein was abolished on deletion of residues 71–82. Moreover, when isolated from rest of the structure, this 12 amino acid segment (residues 71–82) self-polymerize to form highly toxic amyloid fibrils and promote fibrillization of full-length human α-synucelin *in vitro*. Interestingly, highly homologous human β-synuclein which lacks residues 74–84 does not aggregate *in vitro* and not found in Lewy bodies^26–30^. Besides, another closely related human γ-synuclein possess this critical region and forms fibrils like α-synuclein^61^. These results indicate the significance of putative 12-residue core domain within the NAC region in fibril formation. A research group compared the *in vitro* aggregation properties of α-, β-, and γ-synuclein^61^. They found that γ-synuclein fibril formation occurs by a nucleation-dependent mechanism as in α-synuclein. However, γ-synuclein is intrinsically less fibrillogenic and fibril formation takes longer time than α-synuclein. On the other hand, β-synuclein completely failed to fibrillize even under accelerated conditions for several weeks. They concluded that region between amino acids 72 and 84, which is lacking in β- but not γ-synuclein, may contain critical fibrillation determinators^61^.

In our WT simulations on 2N0A, an α-strand (α_R_α_L_α_R_) structure was observed in the region 72–74 for systems **2** (2N0A, WT, Neutral pH, 498 K) and **4** (2N0A, WT, Low pH, 498 K) which were simulated at high temperature. For determining α-strand conformation, dihedral angles were monitored throughout the simulation and plotted. Variation in the dihedral angles of residues 72–74 for the systems **2** and **4** is plotted in Supplementary Figs S1 and S2, respectively. Occupancy of α-strand during 20 ns simulation was found to be 14.84 and 10.85% for systems **2** and **4**, respectively. The position of α-strand within sequence is correlated with the most amyloidogenic regions as determined experimentally^26–30^. Furthermore, these results are in agreement with previous experimental study which investigated the effect of pH and temperature on the human α-synuclein structure and fibrillation^31^. A reversible transition of α-synuclein into a partially folded intermediate was induced by either a reduction in pH or an elevation of temperature. The protein was partially folded under the low pH or high temperature conditions. Changes in the protein environment affect its charge and hydrophobic properties. Low pH minimizes the high net charge of this protein, thereby reduce intramolecular charge-charge repulsion and permit hydrophobic-driven collapse to a partially folded intermediate. High temperature provide a stronger hydrophobic driving force for folding.

The α-strand structure (region 72–74) was not observed for system **1** (2N0A, WT, Neutral pH, 300 K). This is supported by former studies which suggested that such structures are rare under normal physiological conditions^4,5^. Absence of this strand in system **3** (2N0A, WT, Low pH, 300 K) could be due to short simulation time (20 ns). Familial point mutation namely A30P, A53T and E46K which are located in the N-terminal region are reported to influence misfolding, aggregation and fibrillation of α-synuclein^62–65^. However, effect of these PD-related point mutations on the folding behavior of this protein is unclear. Experimental studies revealed that A30P, A53T and E46K exerts strong, modest and subtle influences on local structural propensity, respectively^66,67^. We performed MD simulations on these mutants to inspect their influence on the α-strand formation. Similar to WT systems **2** and **4**, mutant system **5** (2N0A, A30P, Neutral pH, 300 K) showed α-strand (α_R_α_L_α_R_) conformation for the residues 72–74. Furthermore, it was found that α-strand conformation was extended to residue 75 in presence of A30P mutation. Consequently, a four-residue α-strand (α_R_α_L_α_R_α_L_) conformation was observed with high occupancy of 44.28% during 20 ns MD simulation. Variation in the dihedral angles of residues 72–75 for the system **5** is plotted in Supplementary Fig. S3. The α-strand structure couldn’t be observed for A53T and E46K in the short simulation time of 20 ns.

Mutations involving the NAC region such as A30P/A56P/A76P inhibit fibril formation^38^. We performed MD simulation on system **8** (2N0A, A30P/A56P/A76P, Neutral pH, 300 K) to explore the effect of this triple proline mutation on the α-strand formation. During 20 ns simulation, no α-strand structure was observed in the region 72–74 for system **8**. Additionally, we mutated central α-strand residue from Gly73 to proline (G73P) in system **9** (2N0A, G73P, Neutral pH, 300 K). This system also failed to show α-strand structure in the region 72–74. These results demonstrate that occurrence of α-strand within the NAC region (residue 72–74) has substantial importance in the aggregation of α-synuclein.

Phosphorylation of Ser-129 is reported to inhibit fibril formation^39^. We carried out simulations on phosphorylation-mimicking mutation S129E system **10** (2N0A, S129E, Neutral pH, 300 K) and phosphorylated Ser-129 system **11** (2N0A, Ser(P)-129, Neutral pH, 300 K). *In vitro* studies demonstrated that the phosphomimic S129E do not reproduce the effect of phosphorylation on the structural and aggregation properties of α-synuclein and exhibits similar aggregation properties as the WT^39^. Our results are in agreement with the in *vitro* evaluations as both phosphomimic system **10** (2N0A, S129E, Neutral pH, 300 K) and WT system **1** (2N0A, WT, Neutral pH, 300 K) which were simulated under similar conditions didn’t show α-strand structure (region 72–74) during 20 ns MD simulations. Besides, no appearance of α-strand structure in the phosphorylated system **11** (2N0A, Ser(P)-129, Neutral pH, 300 K) indicates that phosphorylation of Ser-129 suppress the assembly of α-synuclein.

A recent paper described that amino-terminally acetylated α-synuclein adopts compact conformations under physiological cell conditions. Such conformations shield aggregation-prone residues of NAC region from exposure to the cytoplasm and counteracts aggregation^68^. Our simulation on N-terminal acetylated system **12** (2N0A, ACE-N-terminal, Neutral pH, 498 K) also demonstrated that acetylation of N-terminal significantly reduced the number of α-strand structures. Occupancy of α-strand (region 72–74) during 20 ns simulation was found to be 2.50% for system **12**. Variation in the dihedral angles of residues 72–74 for the system **12** is plotted in Supplementary Fig. S4. Under the similar simulation conditions, non-acetylated system **2** (2N0A, WT, Neutral pH, 498 K) showed 14.84% occupancy. This demonstrate that N-terminal acetylation slows down the fibrillation.

Similar to 2N0A, simulations on 2KKW also showed α-strand (α_R_α_L_α_R_) structure in the region 72–74 for systems **13** (2KKW, WT, Neutral pH, 498 K) and **14** (2KKW, WT, Low pH, 498 K). However, 2KKW systems showed comparatively lower occupancy of α-strand than 2N0A systems. Occupancy of α-strand during 20 ns simulation was found to be 0.50 and 9.20% for systems **13** and **14**, respectively. Variation in the dihedral angles of residues 72–74 for the systems **13** and **14** is plotted in Supplementary Figs S5 and S6, respectively. Lower occupancy of 2KKW (micelle-bound state) systems as compared to 2N0A (fibril state) systems indicates that fibril state produce relatively more α-strand structures.

### α-strand appearance time

**Figure 3:**
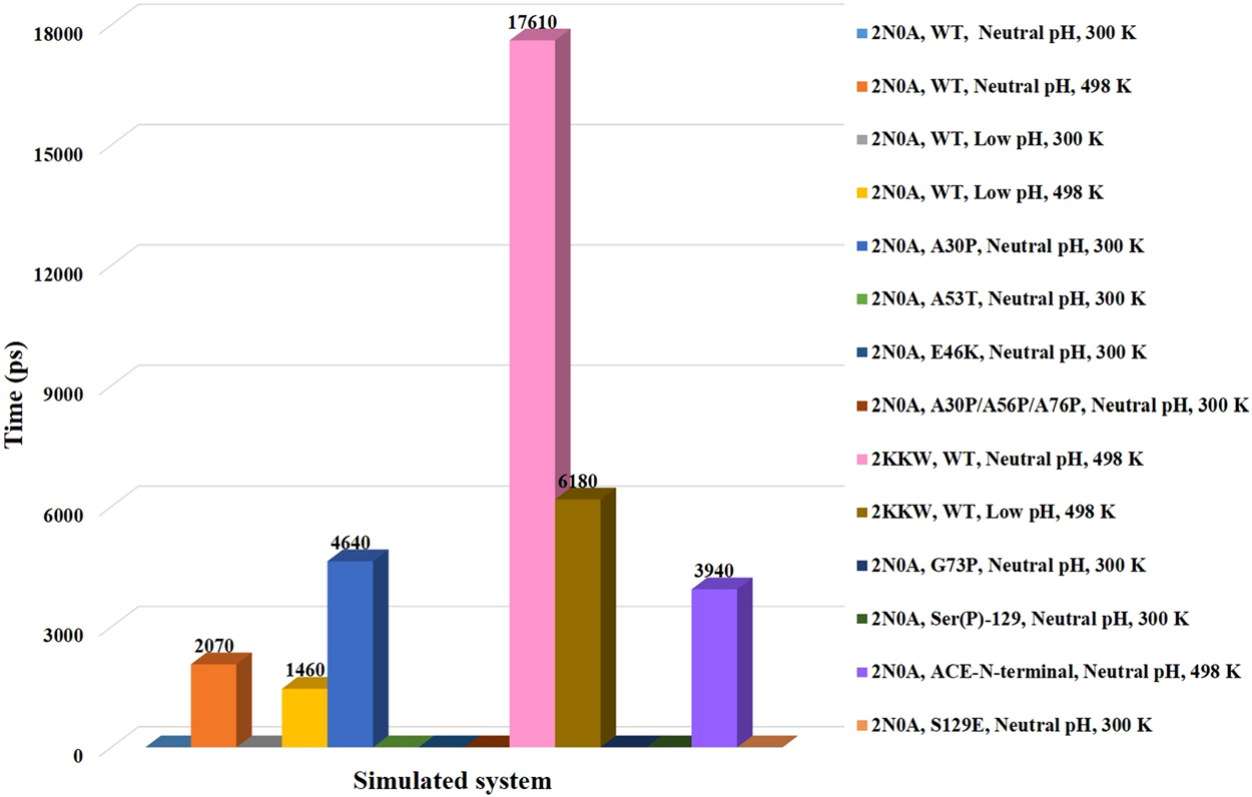
Comparison of α-strand appearance time in the region 72–74 for the simulated systems.

We examined the α-strand appearance time for the simulated systems (Fig. 3). As discussed before, α-strand was not observed for systems **1** (2N0A, WT, Neutral pH, 300 K), **3** (2N0A, WT, Low pH, 300 K), **6** (2N0A, A53T, Neutral pH, 300 K), **7** (E46K, Neutral pH, 300 K), **8** (2N0A, A30P/A56P/A76P, Neutral pH, 300 K), **9** (2N0A, G73P, Neutral pH, 300 K), **10** (2N0A, S129E, Neutral pH, 300 K) and **11** (2N0A, Ser(P)-129, Neutral pH, 300 K) in the region 72–74. Simulation time corresponding to the first appearance of α-strand structure in the region 72–74 was compared for systems **2** (2N0A, WT, Neutral pH, 498 K), **4** (2N0A, WT, Low pH, 498 K), **5** (2N0A, A30P, Neutral pH, 300 K), **12** (2N0A, ACE-N-terminal, Neutral pH, 498 K), **13** (2KKW, WT, Neutral pH, 498 K) and **14** (2KKW, WT, Low pH, 498 K). MD simulation results demonstrated high temperature was important factor for the α-strand formation in WT α-synuclein. In case of systems **2** (neutral pH) and **4** (low pH) α-strand first appeared at 2070 and 1460 ps, respectively. The α-strand formation occurred faster at low pH as compared to neutral pH. This is in agreement with pH-induced partial unfolding of amyloid proteins leading to α-sheet structure^4,48^. First α-strand appeared at relatively longer simulation time (4640 ps) for mutant system **5** (A30P), as this system was simulated at neutral pH and normal temperature. N-terminal acetylated system **12** showed first α-strand at 3940 ps. Under the similar conditions, WT system **2** showed first α-strand in slightly shorter time (2070 ps). This is in accordance with a previous *in vitro* study which reported that N-terminal acetylation delays the fibril assembly of α-synuclein^68^. The first α-strand appeared at 17610 and 6180 ps for systems **13** and **14**, respectively. Delayed appearance of α-strand in 2KKW systems as compared to 2N0A systems suggested that fibril state conformation (2N0A) folds more rapidly into intermediate α-strand structure as compared to the micelle-bound single conformation (2KKW). This is supported by the nucleation-dependent polymerization process which stated that fibril-dependent secondary pathways are much more rapid than primary nucleation process where a fibril nucleus is formed from the monomers^56–59^.

### Transition from β-sheet to α-helix region

**Figure 4:**
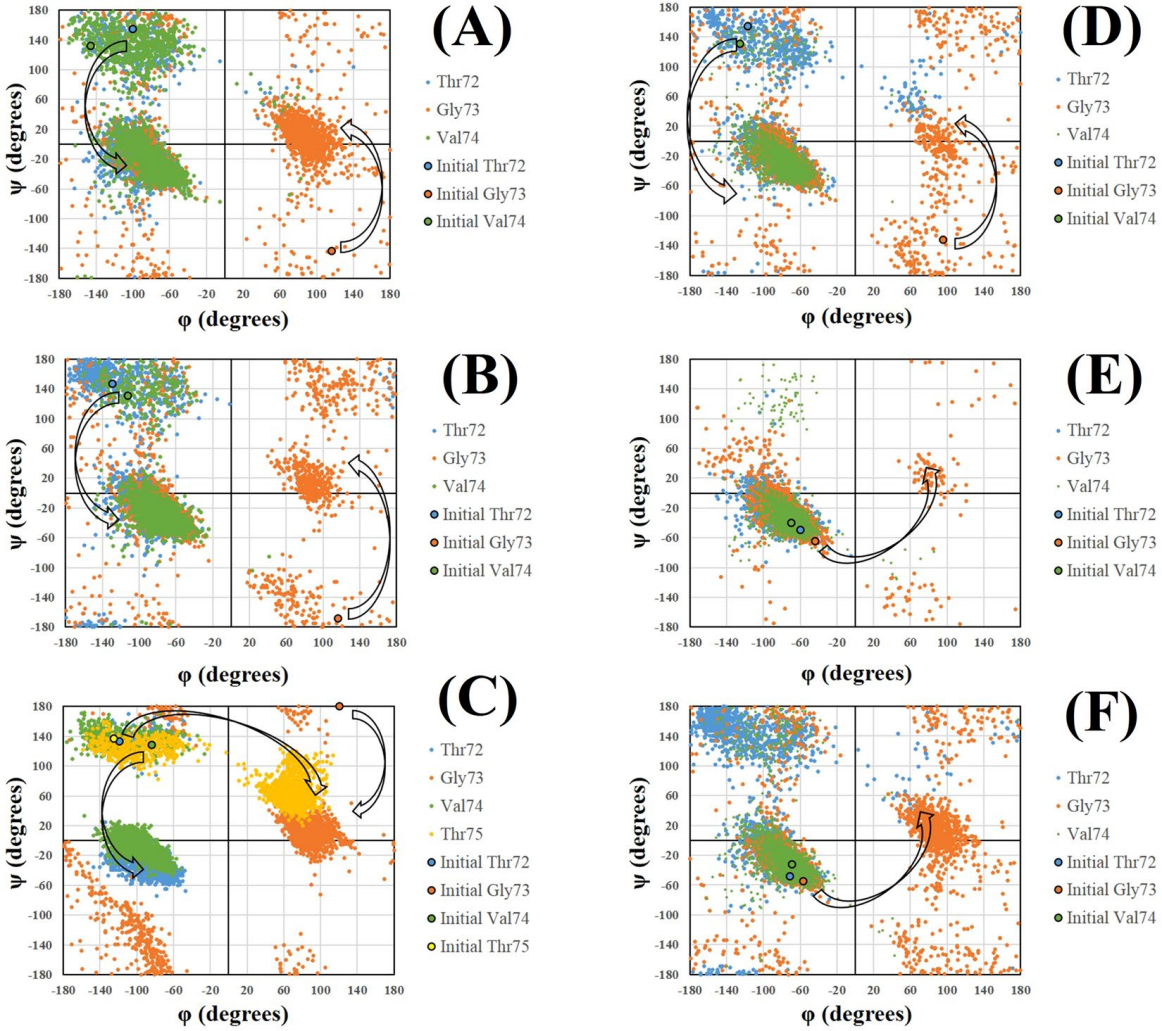
Distribution of dihedral angles for systems **2** (**A**), **4** (**B**), **5** (**C**), **12** (**D**), **13** (**E**) and **14** (**F**) in the region 72–74. Transition of φ and ψ angles for α-strand formation. Initial refers to the dihedrals of particular residue at the beginning of the production run.

Ramachandran plots for 2N0A systems in Fig. 4A–D demonstrate that residues 72 and 74 underwent transition from β-sheet region to the right-handed α-helix region for the systems **2**, **4**, **5** and **12**. Besides, residue 73 displayed transition to left-handed α-helix region from its diverse initial positions. Furthermore, residue 75 showed transition from β-sheet region to left-handed α-helix region in case of mutant system **5** (Fig. 4C). Ramachandran plots for 2KKW systems in Fig. 4E,F show the transition of residue 73 from right-handed to left-handed α-helix region. Sequential transition of backbone dihedral angles of the residues produced the α-strand structure.

### Peptide-plane flip

**Figure 5:**
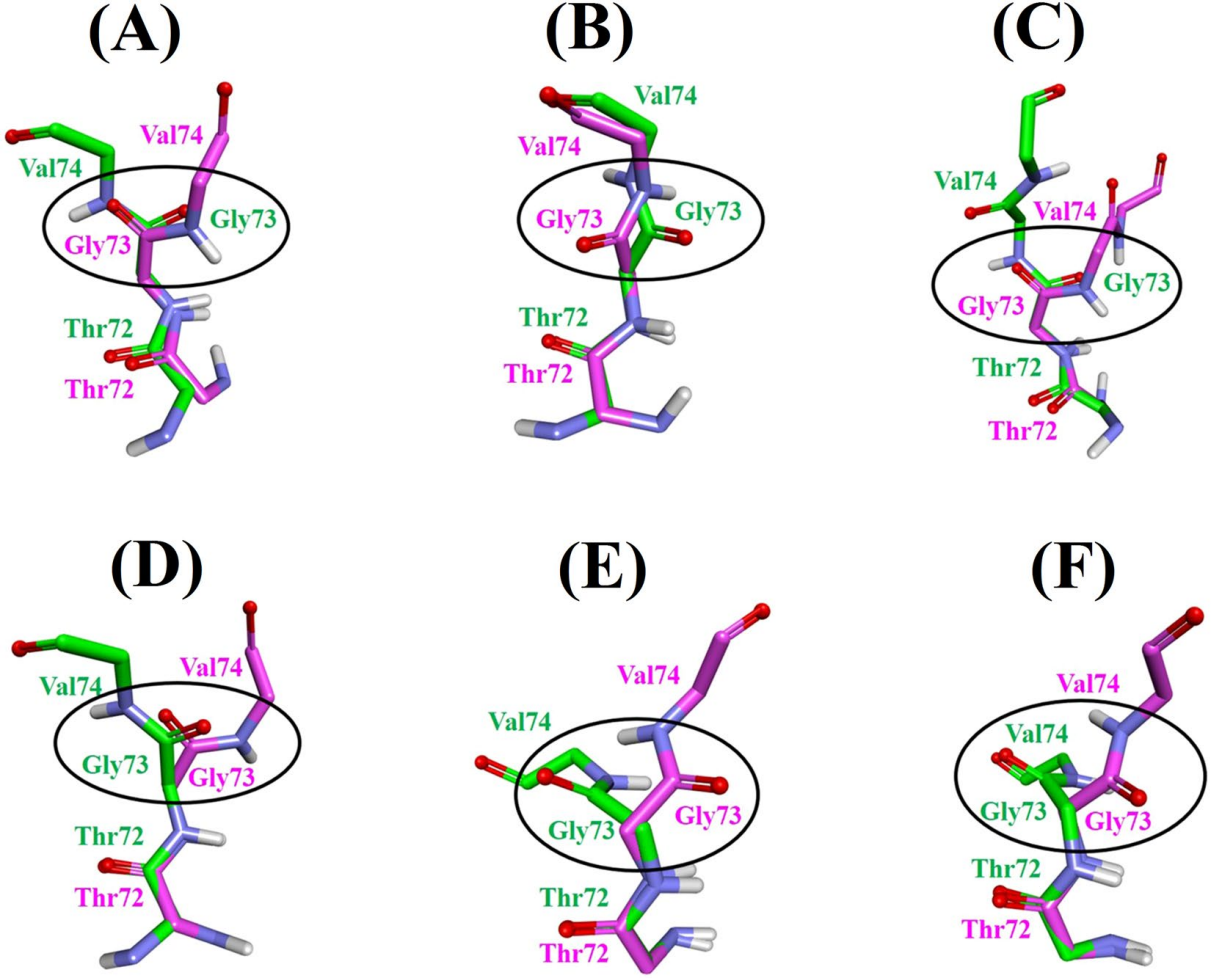
Peptide plane flipping. (**A**) System **2** (2N0A, WT, Neutral pH, 498 K), (**B**) System **4** (2N0A, WT, Low pH, 498 K), (**C**) System **5** (2N0A, A30P, Neutral pH, 300 K), (**D**) System **12** (2N0A, ACE-N-terminal, Neutral pH, 498 K), (**E**) System **13** (2KKW, WT, Neutral pH, 498 K) and (**F**) System **14** (2KKW, WT, Low pH, 498 K). The main chain atoms of the α-strand residues are displayed whereas side chain and non-polar hydrogen atoms are not displayed for clarity. First α-strand conformation is represented by pink stick model while initial conformation at the beginning of the production run is shown by green stick model. Flip of CO-NH plane is highlighted.

As presented in Fig. 4, transition to α-strand region happens sequentially *via* individual transition of backbone dihedral angles instead of all at once in a concerted manner. Such transitions involve rotation of peptide-plane with slight change in side chain orientation and can occur at both physiological and high temperature. However, transition is far slower at physiological temperature and need a longer simulation time^4^. Our results also support the statement as α-strand was observed predominantly for high temperature simulations during 20 ns MD simulation. Peptide-plane phenomenon has been explained in detail elsewhere^6,69^. Peptide-plane flips back if the environment is nonconductive to the transition^4^. In compliance with previous studies^4,6,7^, α-strand formed in our simulation was very dynamic as the peptide-plane flipped back and forth throughout the course of the simulation. As a result of flipping, carbonyl and amino groups are aligned in the α-strand and two complementary partially charged interfaces are formed. In our study, α-strand appeared in the region 72–74 for systems **2** (2N0A, WT, Neutral pH, 498 K), **4** (2N0A, WT, Low pH, 498 K), **5** (2N0A, A30P, Neutral pH, 300 K), **12** (2N0A, ACE-N-terminal, Neutral pH, 498 K), **13** (2KKW, WT, Neutral pH, 498 K) and **14** (2KKW, WT, Low pH, 498 K). For each system, we superimposed the first α-strand and initial conformation at the beginning of the production run to visualize the peptide plane flip. As can be seen in Fig. 5, peptide-plane of Gly73-Val74 underwent flipping in all the α-strand (α_R_α_L_α_R_) forming systems. Additionally, rotation of Val74-Thr75 peptide-plane in system **5** (2N0A, A30P, Neutral pH, 300 K) lead to the extended α-strand (α_R_α_L_α_R_α_L_) formation.

### Evidence for α-strand consisting of Thr, Gly and Val segment in the native proteins

In MD results, we observed α-strand structure for residues 72–74 which corresponds to Thr, Gly and Val amino acids, respectively. Since these are computational results, it is important to know whether such structure exist in experimentally determined protein structures. We searched PDB in order to find available structures where segment comprising of Thr, Gly and Val amino acids are present in α_R_α_L_α_R_ conformation, respectively. Similar to our simulation results, there are 322 PDBs in this database that exhibit α-strand structures. Moreover, in these 322 PDBs, there are 637 occurrences of α-strands. These PDBs support the α-strand (α_R_α_L_α_R_) conformation displayed by Thr, Gly and Val segment (residues 72–74) during MD simulation of α-synuclein. The α-strand structure in the region 72–74 was not observed in any available PDBs of α-synuclein. Hence, present work offers novel structural insights about this amyloid protein. Such information could be useful in understanding its aggregation mechanism.

### Proposed aggregation mechanism of α-synuclein

Traditional experimental methods often cannot unveil protein folding and unfolding routes or intermediate states. By contrast, computational methods enable the observation of such events and play crucial roles in probing the intermediates. MD simulation is a powerful computational tool and has been extensively used for several years to study the structural and dynamic properties of the proteins. MD simulations have shed an interesting light on the early events of protein folding and aggregation. Unfortunately, MD simulations are computationally intensive and thus often limited to pico-to-nano second timescales depending on the size of the system and computing resources. Protein folding or unfolding may require longer simulation time. Nonphysical temperatures have been suggested to accelerate such transitions^5,50^. Nevertheless, different conformations can be observed in the short besides the long time simulations.

**Figure 6:**
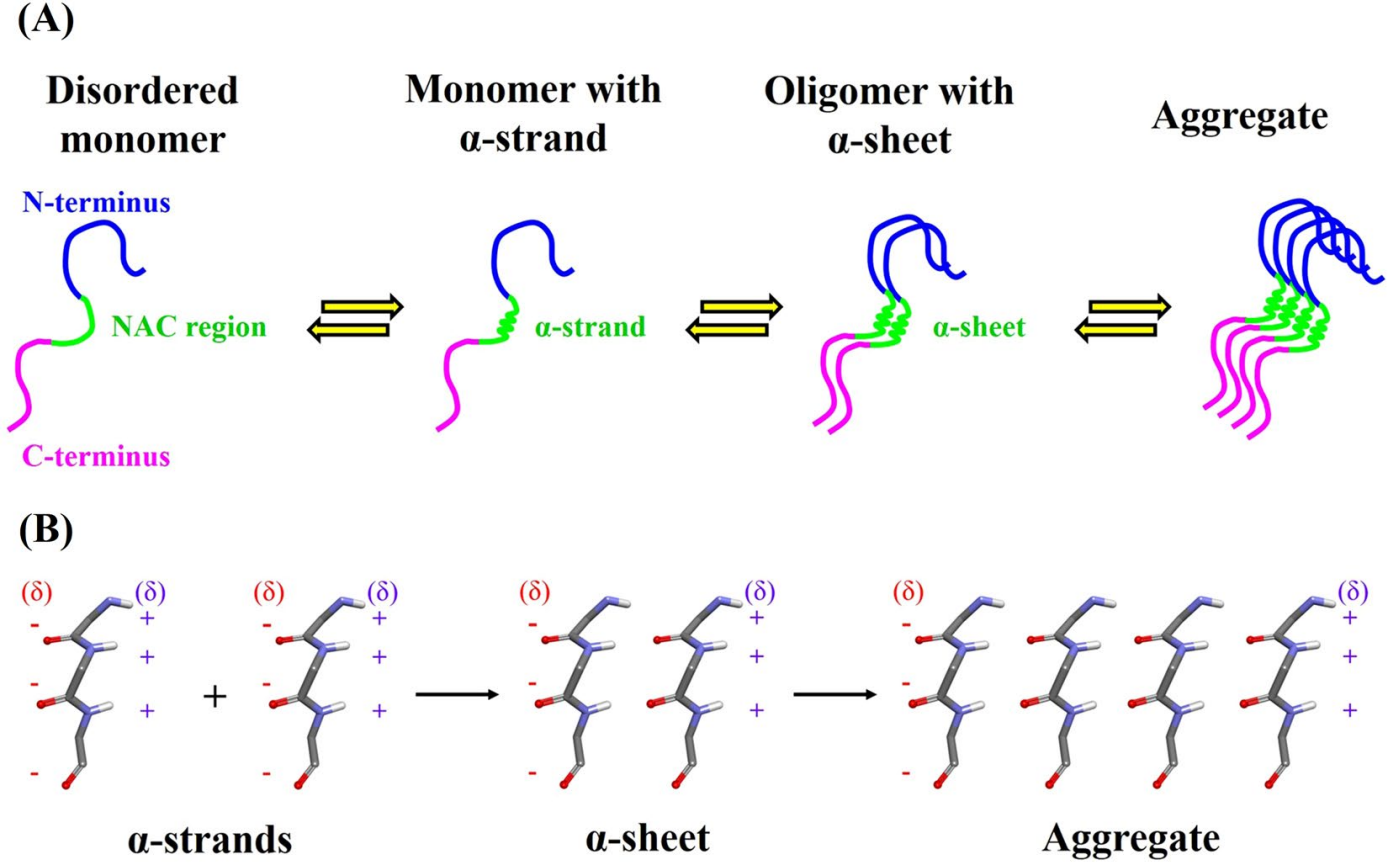
Probable role of α-strand in the aggregation of α-synuclein. (**A**) Suggested stages of aggregation process. N-terminus, NAC region and C-terminus are highlighted in blue, green and pink, respectively. Biophysical studies have confirmed that isolated α-synuclein monomer is intrinsically disordered *in vitro* and aggregation results in the formation of amyloid fibrils. Occasionally, region 72–74 of α-synuclein monomer adopts an α-strand conformation. The α-strands of α-synuclein oligomers interact through hydrogen bonding to form α-sheet structure. Subsequently, α-sheet acts as a possible nucleus that initiate aggregation and fibrillation of α-synuclein. (**B**) A model for α-sheet intermediate where partial charges on the interface are indicated by red (negative charges) and blue (positive charges) colors.

Due to limited computing facilities, we performed MD simulations on α-synuclein monomer for 20 ns and investigated the effects of the selected factors. This simulation time is rather short by current standards as such simulations can suffer from inadequate conformational sampling. Only α-synuclein monomers were simulated and not the dimers as our lab lacks high performance computational resources. Beyond these limitations, our simulations revealed α-strand formation in the critical NAC region (residues 72–74) of α-synuclein. We propose that α-strand structure is possibly involved in the aggregation of α-synuclein. Figure 6A demonstrate how an α-strand structure may act as an intermediate and as an initiation site for oligomerization. This hypothesis is supported by various experimental observations^26–31,42^. However, further experimental studies are required to confirm the role of α-strand/sheet in aggregation and fibrillation of α-synuclein. A number of experimental investigations have provided evidence that α-synuclein exist as an intrinsically disordered monomer^70^. At physiological conditions, α-synuclein monomers remain as disordered structures. However, under typical conditions such as elevated temperature or low pH or point mutations, region 72–74 of monomers occasionally adopts an α-strand conformation. The α-strands of monomers interact through hydrogen bonding to form oligomers with α-sheet structures. Typical feature of the α-sheet is the alignment of carbonyl and amino groups. Partial charges due to this alignment generate two complimentary charged interfaces, as shown in Fig. 6B. The α-sheet structures facilitate self-assembly/aggregation of α-synuclein into soluble protofibrils as a result of attractive forces between the interfaces with opposite charges. Subsequently, transition from α-sheet to β-sheet *via* peptide plane flip causes transformation of soluble cytotoxic protofibrils to insoluble highly ordered amyloid fibrils. Besides, α-sheet may proceed to the mature fibrils. This hypothesis is in agreement with the previous studies which reported a similar theory for various amyloid proteins such as transthyretin, β_2_-microglobulin, lysozyme and prion protein^4,5^. Recently, cryo-electron microscopy (cryo-EM) structure of α-synuclein fibrils was published^71^. In the three-dimensional structure, each α-synuclein(1–121) molecule consisted of eight in-register parallel β-strands. This includes residues 42–46 (β1), 48–49 (β2), 52–57 (β3), 59–66 (β4), 69–72 (β5), 77–82 (β6), 89–92 (β7), and 94-(~102) (β8). Hydrophobic residues including Val74 contributed to the stability of the protofilament^71^. Furthermore, it was suggested that the initial binding event of fibril elongation might be interactions involving residues 74–82. These results further support our findings that residue 72–74 are crucial for fibril formation.

### Strategy for designing α-synuclein aggregation inhibitors

Comprehensive knowledge of the structure and function of a biological target is essential for its inhibitor design. The α-synuclein lacks a well-defined native structure and its aggregation mechanism is ambiguous. X-ray fiber diffraction suggested that insoluble amyloid fibrils are comprised of cross β-sheet structure^4,72^. It is presumed that fibril formation involves a transition from amyloidogenic intermediate to β-sheet structure that is stabilized by intermolecular interactions^4^. Such protein-protein interactions generally involve shallow surfaces and lack well-defined ligand-binding pocket^73^. Accordingly, development of small drug-like inhibitors which could perhaps prevent or reverse the formation of amyloid fibrils is challenging. Hence, researchers are now focusing on peptide-based drug discovery. This includes rational design of short synthetic peptides capable of inhibiting α-synuclein aggregation and design of β-sheet breakers which can bind and isolate β-sheet precursors^74,75^. Nowadays, short peptides can be rapidly and economically synthesized by chemical means and can be modified to improve binding properties. Degradation of peptides is anticipated to be less toxic as compared to synthetic small organic molecules. Various research groups are currently involved in designing peptides which possess the ability to bind to a specific region of α-synuclein and disrupt its aggregation^75–77^.

In the present study, we have proposed an α-synuclein aggregation mechanism on the basis of the α-sheet/strand structure. Suggested hypothesis is supported by previous experimental and modeling studies^4,5,26–31,42^. According to the proposed mechanism, initially α-strand formation occurs in the NAC region (residue 72–74) of α-synuclein monomer. This strand is likely to form α-sheet structure in the oligomers. Afterwards, α-sheet acts as a promising nucleus that initiate and promote aggregation and fibrillation of α-synuclein. We think that α-strand/sheet formation within the NAC region has substantial importance and could be exploited for the rational design of PD drugs. Recently, various anti-α-sheet peptides were reported to inhibit amyloid fibril formation and its associated cytotoxicity^8–11^. Herein, we introduce α-strand/sheet forming region (residue 72–74) of α-synuclein that could be targeted by designed anti-α-sheet peptides. This approach may serve as a novel promising neuroprotective strategy that could aid alleviate PD and its symptoms.

### Topological water network analysis

Proteins are strongly influenced by the surrounding water. Experiments as well as computer simulations revealed that water has a substantial impact on structure, stability, dynamics and function of a protein. Several theoretical studies have explored the effect of water molecules on the structural properties of proteins. However, role of water in the protein folding or unfolding transition mechanism is not well-known^78^. Our research group has previously predicted kinase selectivity on the basis of water-ring network analysis^79,80^. Here, we examined the role of the water network in the α-strand formation within the NAC region of α-synuclein. We focused on the small water-ring network namely three-membered water-ring for the topological water network (TWN) analysis. The water molecules within 15 and 20 Å radius sphere of α-strand residues were obtained from MD trajectory. We calculated the number of three-membered water rings around the α-strand residues and subsequently compared the simulated systems. TWN analysis results are summarized in Table 2. It is evident from the table that total average number of three-membered water rings (*N*) formed around α-strand structures were comparatively lower than the non-α-strand structures for the neutral pH simulations. The *N* values of non-α-strand structures for the system **2** (2N0A, WT, Neutral pH, 498 K) within 15 and 20 Å of residues 72–74 were 44.47 and 131.14, respectively. On the other hand, for the same system, α-strand structures showed *N* values of 40.20 and 123.30 within 15 and 20 Å of region 72–74, respectively. Similarly, *N* values of non-α-strand and α-strand structures for the system **5** (2N0A, A30P, Neutral pH, 300 K) were 31.76 and 27.56, respectively, within 15 Å of residues 72–75 while *N* values were 97.62 and 91.62, respectively, within 20 Å radius. The *N* values of non-α-strand structures for the system **12** (2N0A, ACE-N-terminal, Neutral pH, 498 K) within 15 and 20 Å of residues 72–74 were 14.86 and 41.28, respectively. For the same system, α-strand structures showed *N* values of 10.38 and 36.07 within 15 and 20 Å of region 72–74, respectively. The *N* values of non-α-strand and α-strand structures for the system **13** (2KKW, WT, Neutral pH, 300 K) were 12.75 and 9.53, respectively, within 15 Å of residues 72–74 whereas *N* values were 33.17 and 31.58, respectively, within 20 Å radius.

**Table 2:** Comparison of the average number of three-membered water-rings in the absence and presence of the α-strand structure.

System	Non-α-strand structure						α-strand structure				
	TWN	Thr72	Gly73	Val74	Thr75	Total (*N*)	Thr72	Gly73	Val74	Thr75	Total (*N*)
System **2** (2N0A, WT, Neutral pH, 498 K)	15 Å	15.20	14.75	14.52		44.47	13.26	13.16	13.78		40.20
	20 Å	44.23	43.67	43.24		131.14	40.49	40.65	42.16		123.30
System **4** (2N0A, WT, Low pH, 498 K)	15 Å	12.93	12.53	13.02		38.48	13.18	13.75	13.96		40.89
	20 Å	40.66	40.47	41.38		122.51	41.00	42.41	43.48		126.89
System **5** (2N0A, A30P, Neutral pH, 300 K)	15 Å	8.34	8.62	7.75	7.05	31.76	7.18	7.53	6.69	6.16	27.56
	20 Å	25.28	25.49	23.89	22.96	97.62	23.51	24.06	22.23	21.82	91.62
System **12** (2N0A, ACE-N-terminal, Neutral pH, 498 K)	15 Å	4.72	5.02	5.12		14.86	3.66	3.41	3.31		10.38
	20 Å	13.66	13.82	13.80		41.28	11.67	12.23	12.17		36.07
System **13** (2KKW, WT, Neutral pH, 300 K)	15 Å	4.75	3.75	4.25		12.75	3.31	2.92	3.30		9.53
	20 Å	10.67	11.25	11.25		33.17	10.50	10.33	10.75		31.58
System **14** (2KKW, WT, Low pH, 300 K)	15 Å	4.51	4.24	3.97		12.72	4.92	4.38	4.01		13.31
	20 Å	12.89	12.55	12.41		37.85	13.78	13.67	13.54		40.99

On the contrary, for the low pH simulation, α-strand structures showed relatively higher *N* values as compared to the non α-strand structures. The *N* values of α-strand structures for the system **4** (2N0A, WT, Low pH, 498 K) within 15 and 20 Å of residues 72–74 were 40.89 and 126.89, respectively. However, non-α-strand structures for the same system showed *N* values of 38.48 and 122.51 within 15 and 20 Å of region 72–74, respectively. The *N* values of non-α-strand and α-strand structures for the system **14** (2KKW, WT, Low pH, 300 K) were 12.72 and 13.31, respectively, within 15 Å of residues 72–74 whereas *N* values were 37.85 and 40.99, respectively, within 20 Å radius. Protonation state of the amino acid residues is effected at the low pH that could influence the hydrogen-bonded cyclic water-ring networks. As discussed in the methodology section, His, Asp and Glu residues were protonated for low pH simulation. Accordingly, overall charge on the α-synuclein was affected (Table 1). This could be the probable reason for the dissimilarity in the TWN analysis for neutral and low pH simulations. To get more insight into exactly how surrounding water molecules contribute to the α-strand formation, further studies are advised. Overall, our TWN analysis demonstrated the influence of water network on the occurrence of α-strand in NAC region of the α-synuclein and suggested that water molecules play an active role in the protein folding or unfolding process.

## Conclusion

Various experiments provide evidence that a partially folded intermediate is responsible for α-synuclein fibril formation under low pH and high temperature conditions. Besides, previous studies on amyloid proteins such as lysozyme, prion proteins, β2-microglobulin and transthyretin stated that α-strand/sheet may represent the intermediate as such structure nicely explains many experimental observations. The α-synuclein is also an amyloid protein that forms fibrils. However, α-strand/sheet structure is not yet reported for α-synuclein. We investigated the occurrence of α-strand/sheet structure in this amyloid protein through all-atom MD simulations under different conditions. Interestingly, α-strand structure was observed for residue 72–74 of α-synuclein monomer. These residues are the part of 12 amino acid segment (residues 71–82) within the NAC region of α-synuclein. This region plays a critical role in both the aggregation and cytotoxicity of α-synuclein. Moreover, when isolated from rest of the structure, this segment (residues 71–82) self-polymerize to form highly toxic amyloid fibrils and promote fibrillization of full-length human α-synuclein *in vitro*. Aggregation and neurotoxicity of α-synuclein is abolished on deletion of residues 71–82. Accordingly, occurrence of α-strand within the NAC region (residue 72–74) of α-synuclein has substantial importance. Furthermore, TWN analysis around α-strand residues indicated that existence of such structure is influenced by surrounding water network. Based on the α-strand structure in the region 72–74, we proposed a plausible aggregation mechanism for α-synuclein that is supported by former experimental findings. According to the suggested mechanism, occurrence of α-strand in the monomer lead to α-sheet formation in the oligomers which initiates and promote aggregation and fibrillation of α-synuclein. Hence, crucial region 72–74 could be targeted by designed anti-α-sheet peptides. Such peptides have been found to inhibit aggregation of various amyloid proteins. This promising strategy could inhibit amyloid fibril formation in α-synuclein and could be beneficial for the therapeutic treatment of PD.

## Supplementary information

Supplementary Information

## Data Availability

No datasets were generated or analyzed during the current study.
