# Supplementary material for: Computational insights into the role of α-strand/sheet in aggregation of α-synuclein
Source: Sci Rep. 2019 Jan 11;9:59. doi: 10.1038/s41598-018-37276-1 (PMC6329781; doi:10.1038/s41598-018-37276-1)
Supplement: Supplementary file 1 — Supplementary Information [file 41598_2018_37276_MOESM1_ESM.docx]

Supplementary Information

**Computational insights into the role of α-strand/sheet in aggregation of α-synuclein**

Anand Balupuri, Kwang-Eun Choi and Nam Sook Kang^*^

*Graduate School of New Drug Discovery and Development, Chungnam National University, Daejeon 305-764, Republic of Korea*


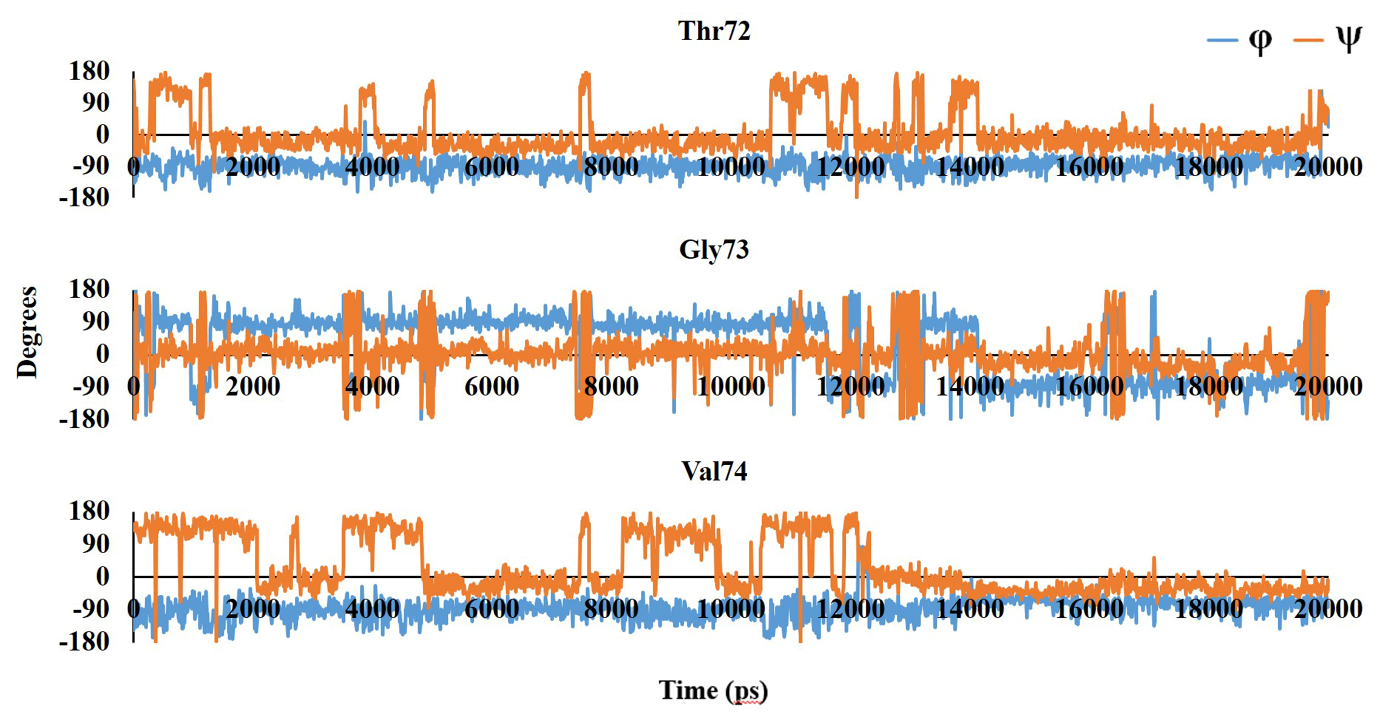


**Figure S1.** Variation in the dihedral angles of residues 72-74 for the system **2** (2N0A, WT, Neutral pH, 498 K) during the 20 ns MD simulation.


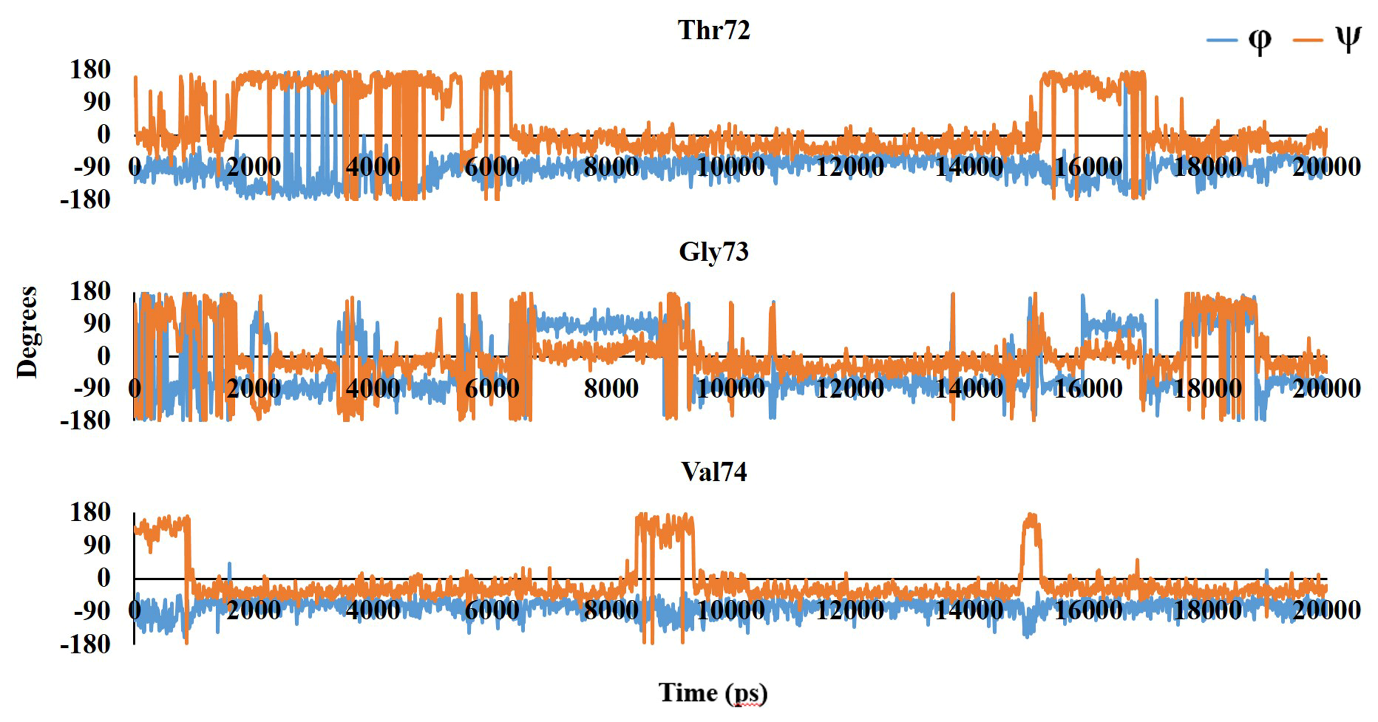


**Figure S2.** Variation in the dihedral angles of residues 72-74 for the system **4** (2N0A, WT, Low pH, 498 K) during the 20 ns MD simulation.


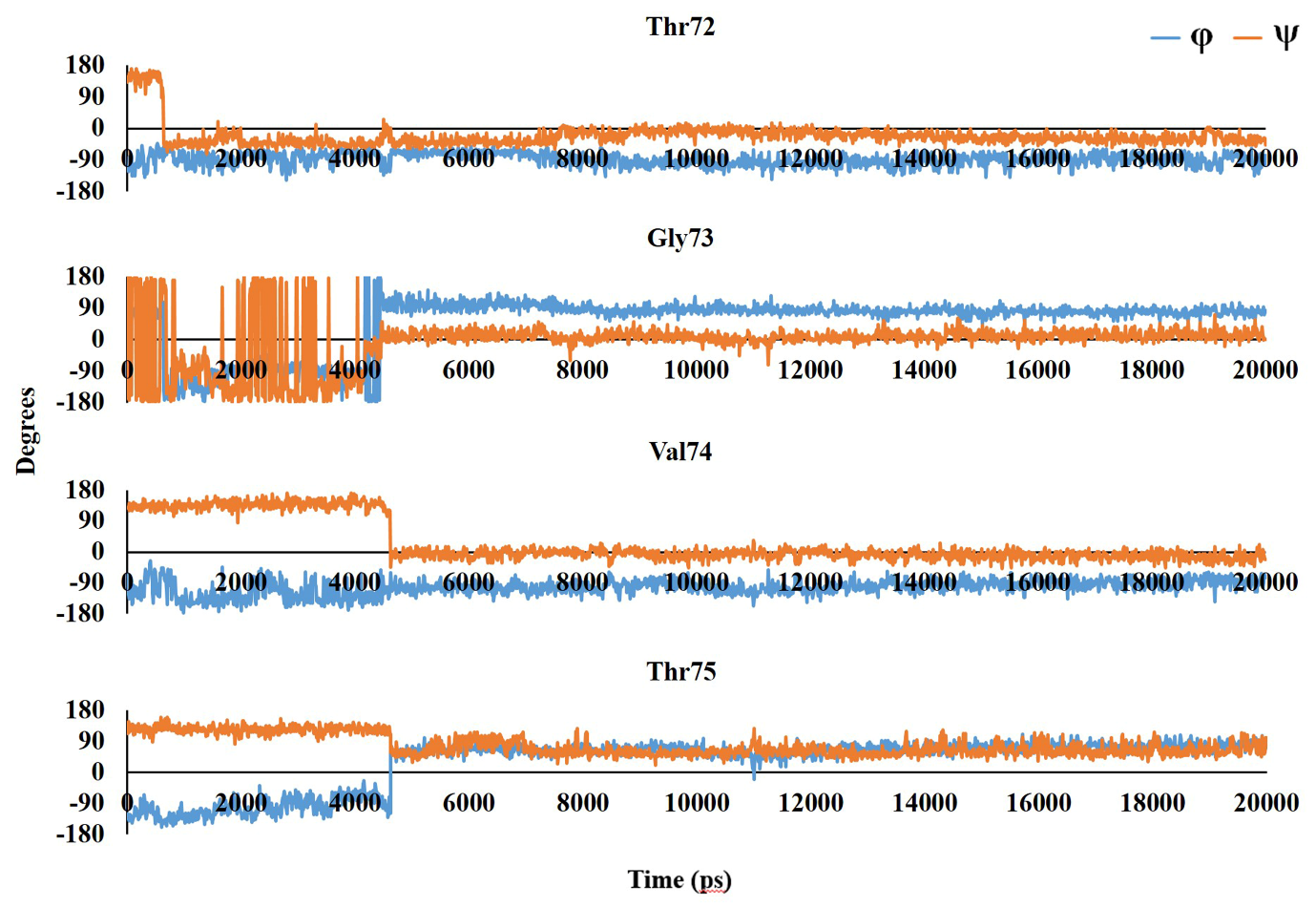


**Figure S3.** Variation in the dihedral angles of residues 72-75 for the system **5** (2N0A, A30P, Neutral pH, 300 K) during the 20 ns MD simulation.


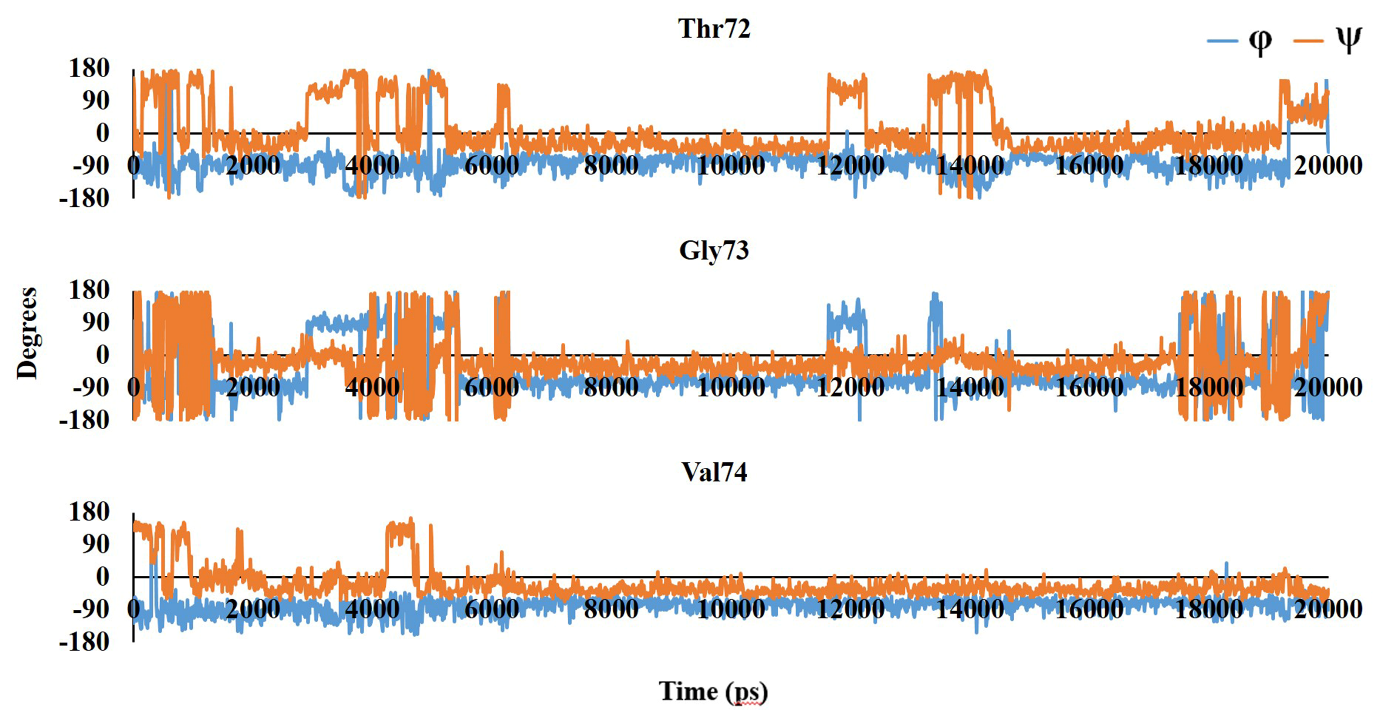


**Figure S4.** Variation in the dihedral angles of residues 72-74 for the system **12** (2N0A, ACE-N-terminal, Neutral pH, 498 K) during the 20 ns MD simulation.


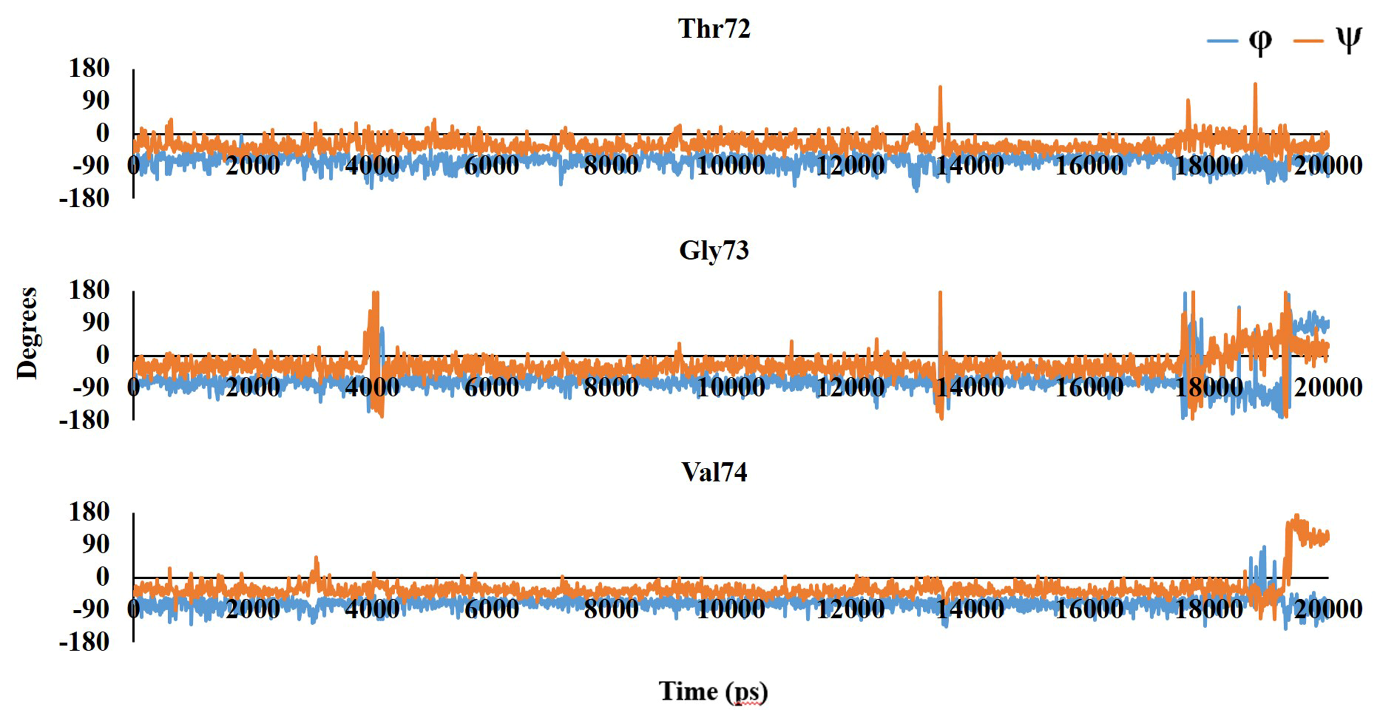


**Figure S5.** Variation in the dihedral angles of residues 72-74 for the system **13** (2KKW, WT, Neutral pH, 498 K) during the 20 ns MD simulation.


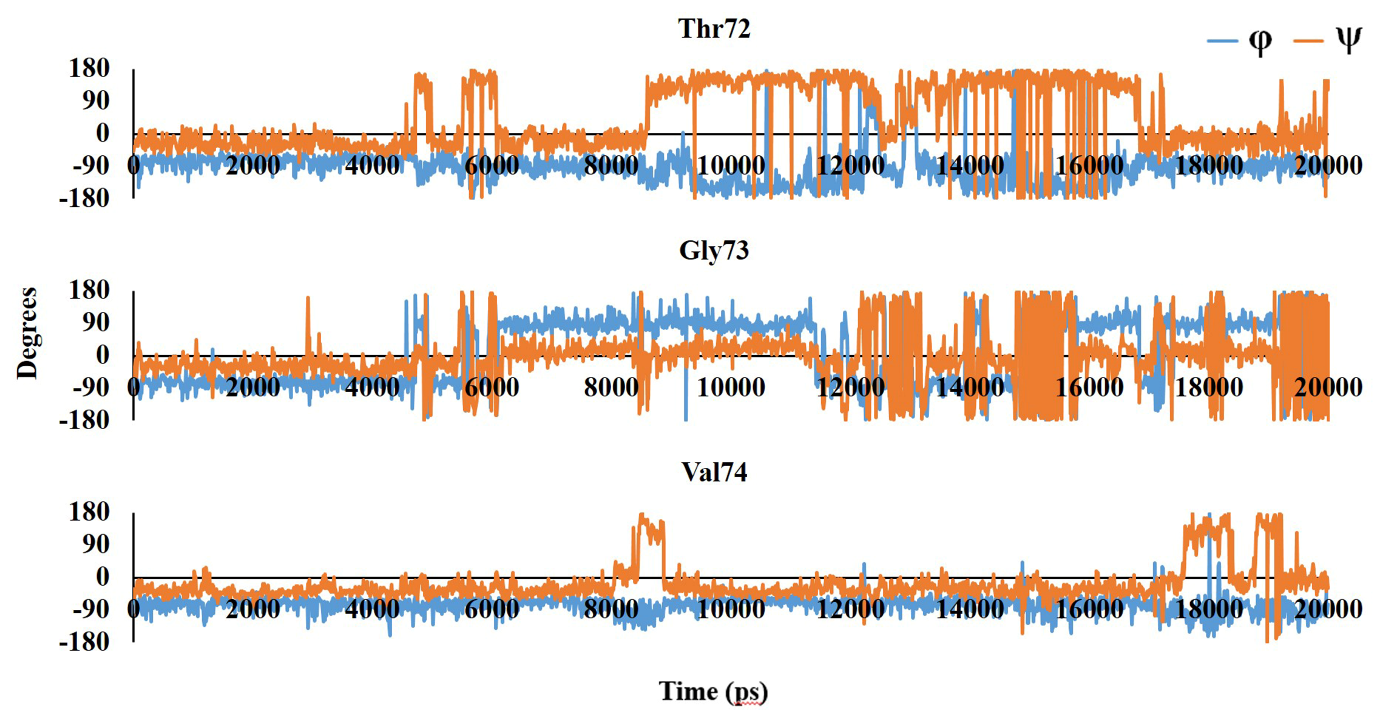


**Figure S6.** Variation in the dihedral angles of residues 72-74 for the system **14** (2KKW, WT, Low pH, 498 K) during the 20 ns MD simulation.
